# Case Series and Literature Review of Up-to-date Surgical Management of Occipital Neuralgia

**DOI:** 10.1055/a-2364-5564

**Published:** 2024-09-09

**Authors:** Seok Joon Lee, Joo Seok Park, Woo Shik Jeong

**Affiliations:** 1 Department of Plastic and Reconstructive Surgery, Asan Medical Center, University of Ulsan College of Medicine, Seoul, Republic of Korea; 2 AMUN Plastic Surgery Clinic, Seoul, Republic of Korea

**Keywords:** migraine surgery, nerve decompression, occipital neuralgia

## Abstract

Nerve decompression is an emerging surgical treatment option for patients with occipital neuralgia. However, limited research is available on the efficacy of this treatment in South Korea. This retrospective study evaluates the efficacy of nerve decompression surgery in patients with chronic migraines, specifically focusing on occipital neuralgia, in South Korea. Between January 2019 and December 2022, six patients diagnosed with occipital neuralgia, who had not responded to conservative treatments, underwent nerve decompression surgery. This procedure, performed under local anesthesia, involved decompression of the greater and/or lesser occipital nerves. Patient data were analyzed for headache frequency and intensity (using the Numeric Rating Scale [NRS]) and the decrease in oral medications needed postsurgery. Results showed significant improvement in headache symptoms postsurgery, with the average preoperative NRS score of 7.9 dropping to 3.7 postoperatively. Additionally, the average number of medications used decreased from 3.2 to 1.3. No significant surgical complications were reported. The study highlights the potential of nerve decompression as an effective treatment for occipital neuralgia, particularly in cases resistant to traditional medical management.

## Introduction

Occipital neuralgia involves severe, piercing pain at the back of the head, neck, and behind the eyes, which is caused by irritation or injury to the occipital nerves that run from the top of the spinal cord to the scalp. 1 Occipital neuralgia can be debilitating and may significantly impact a patient's quality of life. Nerve decompression is an emerging surgical treatment option for patients with occipital neuralgia. However, limited research is available on the efficacy of this treatment in South Korea; therefore, we reported the outcomes of nerve decompression surgery for occipital neuralgia, with an up-to-date review of its surgical management. While the efficacy of migraine surgery has been documented in various studies, limited research is available on the efficacy of this treatment in South Korea. The purpose of this study was to report the outcomes of nerve decompression surgery in patients with chronic migraines in South Korea, with a focus on occipital neuralgia.

## Case

We conducted a retrospective review of patients suffering from chronic migraines who underwent nerve decompression surgery between January 2019 and December 2022 at two medical facilities in South Korea: one tertiary university hospital and one local plastic surgery clinic. All patients were diagnosed with chronic migraines according to the International Classification of Headache Disorders, had occipital headaches, and had failed to respond to conservative treatments. The patients' medical records were reviewed to collect data on age, sex, duration and frequency of migraines, previous treatment history, surgical technique, and outcomes. The primary outcome measures were improvement in headache frequency and intensity (measured by the Numeric Rating Scale [NRS]), and a decrease in the number of oral medications needed for symptom control.

**Fig. 1:**
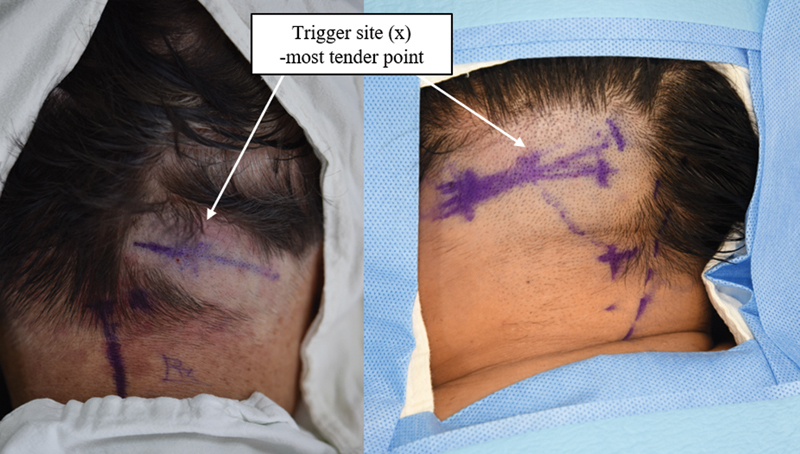
A 5-cm horizontal incision is outlined in the hair-bearing lower occipital region, and the most tender points identified by the patient are marked with an “x.”

**Fig. 2:**
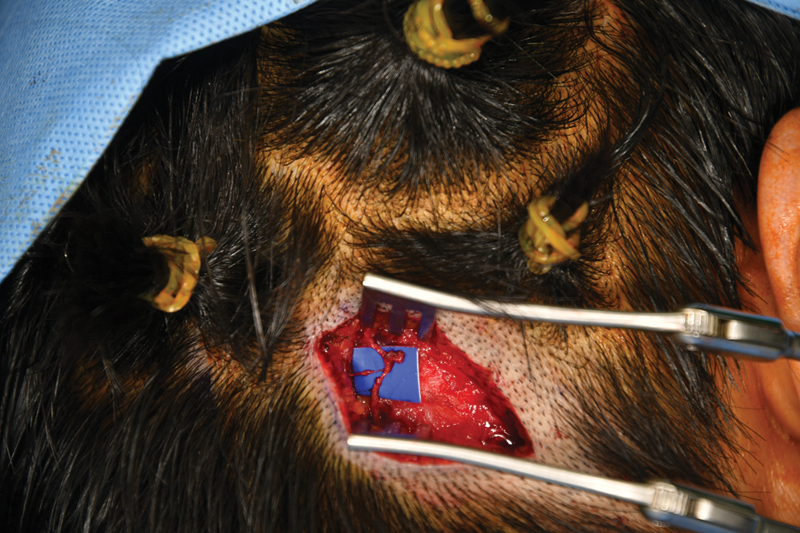
Intraoperative photo of nerve decompression—dissected and released greater occipital nerve.

The surgical technique used in all cases was nerve decompression surgery. The greater occipital nerve and/or lesser occipital nerve were decompressed in all patients suffering from occipital headaches. The surgeries were performed under local anesthesia by an experienced plastic surgeon. The trigger site of the migraine headache was first identified with palpation (“x”). Lidocaine was injected into the target site and an improvement in the headache was typically observed, indicating that the likelihood of headache improvement with surgical decompression was high. The point of the greater occipital nerve, emerging from the trapezius muscle approximately 3.5 cm below and 1.5 cm from the occipital protuberance was marked. An approximate 3.5 cm oblique incision was made along the midpoint between the nuchal line and the lateral edge of the trapezius muscle, positioned a few millimeters caudal to the nuchal line at its most proximal and medial point ( Fig. 1 ). Using this approach, the common tendinous insertion of the sternocleidomastoid and trapezius muscles at the nuchal line, which typically separates into two layers containing the nerve fibers, was dissected to release the nuchal line. This step exposed the greater occipital nerve ( Fig. 2 ). A small medial portion of the semispinalis capitis muscle adjacent to the greater occipital nerve was carefully removed. The dissection then continued distally to free the muscle within the trapezial fascia as the nerve traverses it, extending toward the occiput. If the occipital artery was found impinging on the nerve at the superolateral end, it was dissected and ligated.

This study was approved by the institutional review board of the Asan Medical Center (#2023-0640) and performed in accordance with the principles outlined in the Declaration of Helsinki. The requirement for informed consent was waived because of the retrospective nature of the study, and the analysis used anonymous clinical data. However, informed consent was obtained from two patients specifically for the use of their clinical photographs in this study.

A total of six patients suffering from occipital neuralgia underwent nerve decompression surgery, which involved decompression of the greater occipital nerve and/or lesser occipital nerve. All patients had previously attempted multiple medical treatments, including medication and lifestyle changes but had not experienced significant improvement in their symptoms. Patient demographic data are depicted in Table 1 .

**Table 1:** Demographic details and operation method ( *n* = 6)

*n*	Age	Sex	Smoking	Alcohol	Headache	Decompressed nerve
1	38	F	–	–	Left occiput	Left GON
2	45	M	–	–	Left occiput	Left GON, LON
3	28	M	–	Once per week	Left occiput, forehead	Left LON
4	53	M	Past (6 years)	Once per week	Left occiput	Left GON
5	81	M	Past (20 years)	–	Right occiput, forehead	Right GON, LON
6	40	M	–	Once per week	Both occiput	Left GON

Abbreviations: F, female; GON, greater occipital nerve; LON, lesser occipital nerve; M, male.

Pain scores were evaluated preoperatively, and at 1, 6, and 12 months after surgery. All patients exhibited significant improvements in headache frequency and intensity postsurgery. The average preoperative NRS score was 7.9 (range: 7–9), while the average postoperative NRS score was 3.7 (range: 1–6). The average duration of follow-up was 8.5 months (range: 4–24 months). The number of oral medications used for symptom control decreased from an average of 3.2 medications (range: 2–4) to an average of 1.3 medications (range: 0–2) postsurgery. The difference in frequency and intensity of the headache before and after decompression surgery are presented in Table 2 . None of the patients experienced any significant complications from the surgery.

**Table 2:** Differences before and after decompression surgery (PreOP > PostOP)

*n*	Frequency (per week)		NRS score		Number of oral pills		Follow-up
	PreOP	PostOP	PreOP	PostOP	PreOP	PostOP	
1	3–5	3–5	9	6	4	2	7 months
2	3–5	1–2	7	3	3	2	6 months
3	1–2	0.5	7–8	2–5	3	1	4 months
4	3–5	3	8	4	3	2	4 months
5	5	2–3	8	3–5	2	0	6 months
6	3–5	1–2	8	1–2	4	1	2 years

Abbreviation: NRS, Numeric Rating Scale, Preop: preoperative state; Postop: postoperative state.

## Discussion

Chronic migraine is a severe condition that greatly affects a patient's quality of life. Approximately 10% of the world's population suffers from migraine 2 ; however, this rate varies across country, ethnicity, and age. Chronic migraines are known to be two to three times higher in women than in men, 3 however, five out of six patients were male in our case series. A questionnaire interview in 2012 by Kim et al demonstrated a migraine rate of 6.1% (9.2% in women and 2.9% in men) in South Korea. 4 Primary treatment options for migraines include oral medications for symptom control, such as paracetamol, nonsteroidal anti-inflammatory drugs, and triptans. 5 Despite the availability of numerous medications and therapies for migraines, some patients continue to experience chronic and severe symptoms.

Nerve decompression is an emerging surgical treatment option for patients with medication-refractory chronic migraines. 6 Nerve decompression surgery is based on the theory that chronic compression of peripheral nerves can cause headaches in some individuals. 7 8 By releasing the pressure on the affected nerves, nerve decompression surgery can alleviate the symptoms of chronic migraines. Nerve treatment decompression surgery has been used to treat various types of chronic headaches, including occipital neuralgia. 9 10 11 12 Occipital neuralgia is a neurological condition characterized by paroxysmal, shooting pain centered over the posterior scalp including the occipital nerve region. 1 They are known to be caused by irritation or injury to the occipital nerves that run from the top of the spinal cord to the scalp. Diagnosis is done by clinical examination, but they are known to respond to local anesthetic blocks. 13

**Fig. 3:**
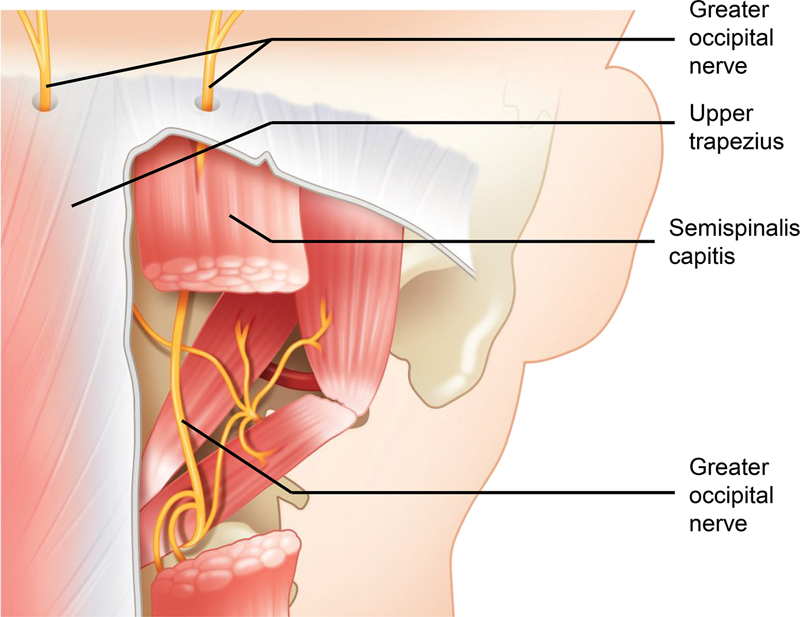
Routes of greater occipital nerves. Fasciotomies to the upper trapezius and semispinalis capitis are often needed for greater occipital nerves to be exposed.

Greater occipital nerve decompression is a useful treatment option for patients who do not respond adequately to medications and physical therapy. The greater occipital nerve is known to be compressed by six known anatomic landmarks, which are obliquus capitis muscle, muscle belly of semispinalis muscle, exit from semispinalis muscle, entrance to trapezial tunnel, insertion of nuchal line, and occipital artery. 14 Our procedure includes releasing the mentioned landmarks, and the course of the greater occipital nerve, trapezius, and semispinalis capitis muscles are depicted in Fig. 3 . The lesser occipital nerve is commonly decompressed together with the greater occipital nerve, which is typically found at the posterior border of the sternocleidomastoid muscle. 15 Contrary to the greater occipital nerve, there are no specific landmarks of compression and its anatomy commonly varies.

Although nerve decompression surgery appears to be a safe and effective treatment option for patients with chronic migraines, 16 not all patients are candidates for this procedure. Nerve decompression surgery should be reserved for patients who have failed to respond to conservative treatments and who have been evaluated by a headache specialist to confirm the diagnosis of chronic migraines. Only migraines of extracranial origin will benefit from nerve decompression surgery. Recently, a prospective cohort study has shown that diagnostic nerve block, where local anesthetic and triamcinolone are injected near the trigger site, can predict symptom improvement after headache surgery. 13 Patients who respond well to nerve block injections will respond well to nerve decompression surgery.

Our study has a limitation in terms of the small sample size, which limits the generalizability of our findings. A larger sample size would allow for a more robust statistical analysis and would enable us to investigate the efficacy of nerve decompression surgery in different subtypes of chronic migraines. Another limitation of our study was the relatively short follow-up period, which limited our ability to assess the long-term outcomes of a nerve decompression surgery. Further studies with larger sample sizes and longer follow-up periods are needed to confirm the efficacy of this treatment in South Korea. Nonetheless, our study contributes to the growing body of literature on migraine surgery and provides valuable insights for clinicians considering this treatment option for their patients.
